# Acceptability and use of waist-worn physical activity monitors in Jamaican adolescents: lessons from the field

**DOI:** 10.1186/s13104-022-06266-y

**Published:** 2023-01-19

**Authors:** Joanne A. Smith, Sara-Lou Christie, Bonny Rockette-Wagner, Lorraine Wilson, Ishtar O. Govia, Keri-Ann Facey, Marshall K. Tulloch-Reid

**Affiliations:** 1 Epidemiology Research Unit, Caribbean Institute for Health Research, The University of the West Indies, 7 Ring Road, Mona, Kingston 7, Jamaica; 2 Department of Epidemiology, University of Pittsburgh, Pittsburgh, PA USA

**Keywords:** Accelerometers, Physical activity, GDAR, Jamaican adolescents

## Abstract

**Objective:**

We report our experience with a validated waist-worn activity monitor in Jamaican adolescents attending urban high schools. Seventy-nine adolescents from the Global Diet and Activity Research (GDAR) study, recruited from 5 urban Jamaican high schools (two coeducational (*n* = 37), two all-female schools (*n* = 32) and one all-boys school (*n* = 10)) were asked to wear Actigraph wGT3X-BT accelerometers for 7 days (24-h), removing the device only when bathing or swimming. They also logged wake up and bed times in an activity diary. Accelerometry was considered valid if at least 4 days with ≥ 10-h monitor wear were recorded. Validity was compared by adolescent demographic and school characteristics. We also reviewed the students’ written feedback on objective physical activity measurement.

**Results:**

Participants, 80.5% female, had a mean age of 15.5 ± 0.8 years with 60% attending schools in low-income communities. Accelerometer return rates were > 98% with 84% providing valid data. Validity did not vary by age group, sex and school setting. While participants were excited about participating in the accelerometer sub-study, commonly reported challenges included monitor discomfort during sleep and maintaining the study diary. Objective measurement of physical activity using 24-h waist-worn accelerometers is feasible and acceptable in Jamaican adolescents.

**Supplementary Information:**

The online version contains supplementary material available at 10.1186/s13104-022-06266-y.

## Introduction

Physical inactivity is one of the four main drivers of the worldwide epidemic of non-communicable diseases (NCDs) [1]. The 2017 Global School Health Survey found that less than 25% of Jamaican adolescents reported at least 60 min of physical activity (PA) in the 7 days preceding the survey [2]. In response to the growing prevalence of NCDs and their risk factors the Jamaica Ministry of Health & Wellness launched the Jamaica Moves Campaign in 2017 [3]. The goal of this campaign is to promote healthy lifestyle behaviours including meeting the World Health Organization (WHO) recommendation for PA.

The Global Diet and Activity Research (GDAR) network was formed in 2017 to focus on upstream determinants of diet and PA particularly in adolescents [4]. Early life PA habits are of particular relevance to the network since physical inactivity during childhood is associated with obesity [5] and heightens the risks for NCDs in adulthood [6]. Moreover, PA habits are usually established in early life, and a better understanding of these habits in adolescence is crucial in abating the NCD epidemic.

Where possible, objective methods of measuring PA such as accelerometers are preferred in youth given their greater prevalence of reporting bias on questionnaire-based methods and lower compliance with completing daily activity logs compared to adults [7, 8]. Recently, there have been important developments in methods specific to accelerometry in youth. However, despite this, there is still very limited use of these devices in research conducted within low and middle income countries (including the Caribbean), particularly among youth [9–11]. Additionally, little is known about acceptability of monitor wear among Caribbean youth [12].

We describe our experience from a feasibility study using a 24-h waist-worn accelerometer in Jamaican adolescents who were part of the GDAR study [4]. Both their ability to adhere to the accelerometer measurement protocol to achieve valid wear time and their experiences wearing the device are presented.

## Main text

### Methods

#### School selection

The larger GDAR study involved five high schools with 229 student volunteer participants from grades 7 to 11, aged 13 to 18 years [4]. Schools were either coeducational (n = 2) or unintegrated (2 all girls and one all boys) and were located in low- or high-income communities in Kingston and St. Andrew, Jamaica’s capital city. Schools were selected based on the land value indices of the neighbouring communities. The data presented are for students enrolled between May 2019 and March 2020 prior to the reporting of the first COVID-19 case in Jamaica. Participants in the accelerometry sub-study were selected from grades 10 and 11.

#### Accelerometry

The Actigraph wGT3X-BT was used to measure physical activity and purchased from the funded project budget. The Actigraph monitors have been validated against doubly labelled water which has been used as the gold standard for measuring physical activity energy expenditure (PAEE) [13–16]. Students were asked to wear the monitor on their waist for 7 continuous days to capture 24-h movement (inclusive of physical activity, sedentary behaviour and sleep). The monitors were initialized by study staff using ActiLife® software v6.13.3 at a sampling rate of 60 Hz. Accelerometry data were downloaded using the ActiLife® software. Measurements were considered valid if there were at least 4 days with 10 h of wear time recorded.

#### Adolescent engagement

A teacher liaison introduced the research team to each class from Grades 10–11 where the study was explained, and interested students provided with consent forms explaining the study to obtain parental approval. After providing written parental informed consent and adolescent assent, GDAR study participants were scheduled to complete study questionnaires and other measures related to the larger study outcomes. On the first visit, if accelerometers were available, participants for the sub-study were fitted with an accelerometer, handed an accelerometer instruction sheet, tip sheet and activity diary. However, if monitors were in use at another school, another visit was scheduled for the sub-study participants.

Additionally, all sub-study participants spent at least five minutes in an individualized session with a member of the research team to demonstrate accurate placement of the activity monitor and correct filling in of their activity diary. Each student attached the monitor and filled in Day 1 of the diary on their own. Participants were instructed to wear monitors on their right hip in line with the arm pit and record wake up and bedtimes in their diaries. Students also received a contact number for project staff.

Follow-up with each student was done through a single reminder WhatsApp® or short messaging service (SMS) text message to encourage consistent wear of the accelerometer. After return of accelerometers and diaries, students completed a brief physical activity questionnaire (Additional file 1) and wrote about their experience with the activity monitor and the diary.

All students who participated were provided with a non-monetary token.

#### Data analysis

The wear time was analyzed using ActiLife® software v6.13.3. The Fisher’s exact test was used to assess statistical significance, with *P*-value ≤ 0.05 indicating significance. A thematic analysis of students’ feedback on wear of the monitors and use of the diaries was conducted.

### Results

Seventy-nine student participants received accelerometers. One participant lost his/her device and another withdrew before completing data collection. Data are therefore presented for 77 students -mean age (± SD) 15.5 ± 0.8 years, 80.5% female. Fifty-four percent of the sub-sample attended unintegrated (single sex) high schools. Fifty-four percent of participants attended schools in low-income settings.

Overall, 84% of monitors downloaded returned records meeting the criteria for valid wear time. Table 1 outlines the characteristics of adolescents according to whether or not they provided valid accelerometer records. No statistically significant differences were observed in the validity of recordings by age-group, sex or school setting (high or low income).

**Table 1:** Demographic characteristics of study participants according to validity^a^ of accelerometry data

	Valid data n (%)	Non-valid data n (%)	*P-*value
Gender			0.1
Male	13 (87)	2 (13)	
Female	52 (84)	10 (16)	
Age group			0.127
≤ 15 years	38 (90)	4 (10)	
≥ 16 years	27 (77)	8 (23)	
School socio-economic status			0.206
Situated in low-income settings	33 (79)	9 (21)	
Situated in high-income settings	32 (91)	3 (9)	

One participant lost his/her accelerometer

^a^Data considered valid if there were more than 4 days of greater than 10 h wear time

The primary reasons of non-valid data from the participants were discomfort from the device, forgetting to replace the monitors after removal, illness during the period of study or a lack of interest in following the accelerometer wear protocol.

#### Experience wearing an accelerometer

The three recurring themes that emerged from review of students’ documented experiences with the monitors were:—*excitement* from participating in the study, *discomfort* wearing the accelerometer and *anxiety* during the period of wear.

Many students reported *excitement* when wearing the physical activity accelerometers. They felt a sense of responsibility being part of a research project. The study was perceived as a positive experience by many. *“Wearing the monitor was a great experience knowing that doing it aids in a physical activity research at UWI* [The University of the West Indies]*” (Student* ≤ *15). “Wearing the monitor made me feel responsible. I wore it right through the day and take it off when I am going to bathe” (Student* ≤ *15).*

Feelings of *discomfort* were noted among students, which were experienced mostly during sleep and less frequently during walking or exercise. *“Mixed feelings, disturbing during dancing activities and sleep time. I am awaken*[ed] *and have to readjust same” (Student* ≤ *15).*

*“I was happy I could participate. Sometimes the belt can be quite uncomfortable. It feels like something piercing into your side even when you adjust it” (Student* ≤ *15).* Several students reported that their comfort levels improved as they got use to wearing the monitors but some reported feeling pain and skin irritation even when the elastic belt was loose. *“At first, it was uncomfortable, but I eventually got use[d] to it. It was an odd experience as I’d never done something like this before, but it wasn’t bad” (Student* ≤ *15).*

Students reported *anxiety* during the study period, particularly from having to remember to replace the device after removing it for various reasons. *“It was stressful because you have to remember to put it back on, but otherwise it was exciting” (Student* ≤ *15). “It was slightly difficult in terms of remembering to put it on after showering…” (Student* ≤ *15). “Sometimes I had to keep it somewhere where I could see it when I took it off” (Student* ≥ *16).*

#### Experience completing an activity diary

Three major themes emerged from students’ feedback about the diaries. These were *ease of use*, *burden* from daily recording and *self-awareness* from reflection on daily routines.

Many students described completing the activity diary as easy and simple. *“The activity diary was a breeze* [easy to use] *and very simple as well as the instructions were clearly stated hence making it an easy task” (Student* ≤ *15). “Completing the diary was the easiest task since all I did was mark date and time” (Student* ≥ *16). “It was very easy to do because I always monitored what I did while I had it on or took if off” (Student* ≤ *15).*

However, some students reported challenges documenting the data required for the diary (wake and bed times). They found the daily recordings as an added task and a burden. *“My experience completing the diary was frustrating because most times I have to remember the times I woke up” (Student* ≤ *15). “It was kind of hard to remember to write in it when I had a lot of school work to do” (Student* ≥ *16). “It was alright to write in the diary in the morning but when it was in the night, I felt sleepy so sometimes I would have to write it down somewhere to remember” (Student* ≥ *16).*

Students commented that the diary heightened their *self-awareness* of their daily routines such as sleeping habits. It also provided empowerment and a sense of responsibility and satisfaction when completed. *“It helped me realized that I don’t get enough sleep and I have to wake up pretty early after the amount of sleep I got.” (Student* ≤ *15) “It gave me a sense of responsibility, in filling out every day.” (Student* ≥ *16) “It was satisfying to help the researchers collect data.” (Student* ≥ *16).*

### Discussion

Although the use of physical activity accelerometers for research among adolescents is novel within the Jamaican context, 84% of accelerometers returned provided valid data. Based on participant responses there was also a high acceptance toward wearing the monitors.

There were a number of challenges reported by adolescents, like *forgetting to wear the devices* and *devices being uncomfortable*. These issues are not unique to Jamaica. Sirard et al. found that high schoolers returned higher percentages of viable data when told they would receive compensation based on the number of complete days of data (96% versus 70% in the control group) [17]. In our study we achieved high levels of acceptance and compliance without the promise of an incentive.

Many students in our study reported discomfort with the accelerometer. Young persons in a study by Kirby et al. also described their devices as uncomfortable or irritating [18]. Thus, manufacturers should therefore consider designing less obtrusive monitors and belts that do not negatively affect sleep. In our study we recommended to participants that wearing devices over a layer of clothing (e.g. undergarments) rather than directly on the skin could lessen the occurrence of skin irritation. The wearing of the device at another location—such as the wrist—may also be less problematic in this age group.

The activity diaries were another challenge for our participants. Sirard et al. reported that use of activity diaries increased wear compliance (85% with the diary compared to 70% in the control group) [17]. This may explain the high rates of valid wear data in our study. It is generally advisable that diaries be easy to complete and not require too much information. In our study, many students found the diaries useful and easy to complete; only a few found it confusing. We found it helpful to provide students with added support through follow-up telephone calls or text messages. While we only contacted participants once while wearing devices, additional support may have made the process of maintaining the diary easier. An electronic diary or app may also be appropriate for teenagers. Lastly, involvement of peers as well as school staff and parents/guardians may have also improved adherence to our accelerometry measurement protocols.

## Limitations

Despite our validation study being relatively large, we may have been underpowered to demonstrate statistically significant differences by student and school characteristics. Additionally, we recruited older volunteers for this sub-study and so adherence to the protocol and use of the instruments may be higher than if this study were to be conducted in a more randomly selected population.

Physical activity accelerometers are an objective approach for monitoring levels of physical activity among urban-dwelling Jamaican adolescents, with 84% of adolescents providing valid data. Lessons learnt from our experience can help other researchers in similar settings implement this approach to physical activity assessment in youth.

## Supplementary Information

**Additional file 1**: Brief physical activity questionnaire.

## Data Availability

The data that support the findings of this study are available from the University of Cambridge, but restrictions apply to the availability of these data, which were used under license for the current study, and so are not publicly available. Data are however available from the authors upon reasonable request and with permission of the University of Cambridge.

## Abbreviations

GDAR: Global Diet and Activity Research
NCD: Non-communicable diseases
PA: Physical activity
WHO: World Health Organization
COVID-19: Coronavirus disease
SMS: Short messaging service
UWI: The University of the West Indies
NIHR: The National Institute for Health and Care Research
NHS: National Health Service
