# Supplementary material for: Acceptability and use of waist-worn physical activity monitors in Jamaican adolescents: lessons from the field
Source: BMC Res Notes. 2023 Jan 19;16:3. doi: 10.1186/s13104-022-06266-y (PMC9849105; doi:10.1186/s13104-022-06266-y)
Supplement: Supplementary file 1 — Additional file 1: Brief physical activity questionnaire. [file 13104_2022_6266_MOESM1_ESM.pdf]

Brief Physical Activity Questionnaire		
	**Question**	**Answer**
**SECTION 1: INTERVIEW DETAILS** [Interviewer to complete]		
1.1	School ID	
1.2	Individual ID	
1.3	Interviewer Name	
1.4	Interviewer ID	
1.5	Consent form signed by participant's parent/guardian	☐ Yes ☐ No
1.5a	Activity monitor received from participant	☐ Yes ☐ No
1.5b	Activity diary received from participant	☐ Yes ☐ No
1.6	Completion	☐ Yes ☐ No
1.7	Visit 2 date	
1.8	Visit 2 time started	
1.9	Visit 2 time ended	
1.10	Main language of interview	☐ English<br>☐ Other ________________________
1.11	Supervisor signature	
1.12	Supervisor date	
1.13	Data entry signature	
1.14	Data entry date	
**SECTION 2: FEEDBACK**		
2.1	Please give a short description of your experience wearing the activity monitor	
2.2	Please give a short description of your experience completing the activity diary	
